# A dual tracer ^68^Ga-DOTANOC PET/CT and ^18^F-FDG PET/CT pilot study for detection of cardiac sarcoidosis

**DOI:** 10.1186/s13550-016-0207-6

**Published:** 2016-06-17

**Authors:** Lars C. Gormsen, Ate Haraldsen, Stine Kramer, Andre H. Dias, Won Yong Kim, Per Borghammer

**Affiliations:** Department of Nuclear Medicine & PET Center, Aarhus University Hospital, Nørrebrogade 44, 8000 Aarhus C, Denmark; Department of Cardiology, Aarhus University Hospital, Palle Juul-Jensens Boulevard 99, 8200 Aarhus N, Denmark

**Keywords:** Cardiac sarcoidosis, PET/CT, Somatostatin receptor imaging, Inflammation, Heart failure

## Abstract

**Background:**

Cardiac sarcoidosis (CS) is a potentially fatal condition lacking a single test with acceptable diagnostic accuracy. ^18^F-FDG PET/CT has emerged as a promising imaging modality, but is challenged by physiological myocardial glucose uptake. An alternative tracer, ^68^Ga-DOTANOC, binds to somatostatin receptors on inflammatory cells in sarcoid granulomas. We therefore aimed to conduct a proof-of-concept study using ^68^Ga-DOTANOC to diagnose CS. In addition, we compared diagnostic accuracy and inter-observer variability of ^68^Ga-DOTANOC vs. ^18^F-FDG PET/CT.

**Methods:**

Nineteen patients (seven female) with suspected CS were prospectively recruited and dual tracer scanned within 7 days. PET images were reviewed by four expert readers for signs of CS and compared to the reference standard (Japanese ministry of Health and Welfare CS criteria).

**Results:**

CS was diagnosed in 3/19 patients. By consensus, 11/19 ^18^F-FDG scans and 0/19 ^68^Ga-DOTANOC scans were rated as inconclusive. The sensitivity of ^18^F-FDG PET for diagnosing CS was 33 %, specificity was 88 %, PPV was 33 %, NPV was 88 %, and diagnostic accuracy was 79 %. For ^68^Ga-DOTANOC, accuracy was 100 %. Inter-observer agreement was poor for ^18^F-FDG PET (Fleiss’ combined kappa 0.27, NS) and significantly better for ^68^Ga-DOTANOC (Fleiss’ combined kappa 0.46, *p* = 0.001).

**Conclusions:**

Despite prolonged pre-scan fasting, a large proportion of ^18^F-FDG PET/CT images were rated as inconclusive, resulting in low agreement among reviewers and correspondingly poor diagnostic accuracy. By contrast, ^68^Ga-DOTANOC PET/CT had excellent diagnostic accuracy with the caveat that inter-observer variability was still significant. Nevertheless, ^68^Ga-DOTANOC PET/CT looks very promising as an alternative CS PET tracer.

**Trial registration:**

Current Controlled Trials NCT01729169.

**Electronic supplementary material:**

The online version of this article (doi:10.1186/s13550-016-0207-6) contains supplementary material, which is available to authorized users.

## Background

Sarcoidosis is an inflammatory disease characterized by non-necrotizing sarcoid granulomas that are often located in the mediastinal lymph nodes and the lungs but may also affect other organs [1]. In up to 25 % of patients, the heart may be involved [2]. Whereas pulmonary sarcoidosis is often self-limiting and spontaneously resolves, cardiac sarcoidosis (CS) causes myocardial inflammation and fibrosis that may ultimately lead to serious heart failure, arrhythmias, and sudden death [3, 4]. Patients with CS should therefore receive high-dose corticosteroid therapy, which has been demonstrated to improve survival [5, 6].

However, cardiac sarcoid lesions are often patchy and located in areas not readily accessible by endomyocardial biopsy. Consequently, histological verification is rare, and sensitivity of the endomyocardial biopsy is also poor (<20 %) [7]. In the absence of a proper diagnostic reference standard, a set of diagnostic criteria laid out by the Japanese Ministry of Health and Welfare (JMHW) has gained widespread acceptance. The JMHW criteria are comprised of various electrocardiographic and imaging abnormalities including cardiac MRI [8] but also accumulation of ^67^Ga citrate in the cardiac region imaged by conventional planar scintigraphic imaging. The ^67^Ga citrate planar scan has since the introduction of the JMHW criteria been surpassed by the superior positron emission tomography (PET) technique, which is likely to replace ^67^Ga citrate scintigraphy as a major criterion for CS. PET scanners have better spatial resolution than conventional gamma cameras and the widely used glucose analogue radiotracer 18-fluoro-2-deoxyglucose (^18^F-FDG) is avidly taken up by the activated macrophages, epithelioid cells, and Langerhans giant cells found in sarcoid granulomas. Thus, ^18^F-FDG PET/CT have been reported to display high sensitivity and specificity for detecting CS [9]. Even though ^18^F-FDG PET/CT has not been formally incorporated into the JMHW criteria for CS, other diagnostic algorithms like those put forward by the Heart Rhythm Society (HRS) [10] include focally increased ^18^F-FDG in areas of the myocardium as a sign of CS.

A major obstacle to successful use of cardiac ^18^F-FDG in diagnosing CS is related to the non-specific nature of the radiotracer. Since ^18^F-FDG is a glucose analogue, it is taken up by all glucose-consuming tissue including the heart, which relies on a mixture of glucose (~30 %) and fatty acids (~70 %) for fuel consumption [11]. Pathological ^18^F-FDG uptake by activated inflammatory cells infiltrating the myocardium may therefore be obscured by physiological ^18^F-FDG uptake by normally functioning cardiomyocytes. In addition, even though myocardial ^18^F-FDG uptake may be suppressed by various pre-scan fasting protocols [12, 13], uptake patterns are often heterogeneous and may mimic pathological uptake [14]. Visual ^18^F-FDG PET/CT image interpretation in patients undergoing diagnostic work-up for suspected cardiac sarcoidosis is therefore very much reliant on the experience of the reading nuclear medicine physician.

A better alternative to the non-specific ^18^F-FDG PET tracer could be somatostatin receptor (SSTR)-targeted radiotracers like ^68^Ga-DOTA-NaI-octreotide (DOTANOC) or ^68^Ga-DOTA-D-Phe-Tyr-octreotide (DOTATOC). Activated inflammatory cells (epithelioid cells, multinucleated giant cells, and some macrophages that are typically found in sarcoid granulomas) have abundant SSTRs on their surface [15], in particular SSTR2A, whereas normal cardiomyocytes do not. ^68^Ga-DOTANOC has a high affinity for both SSTR2 and 5 and are not taken up by the normal myocardium to any significant extent [16], whereas some physiological uptake in the myocardium is observed using ^68^Ga-DOTATOC [17]. Signal-to-noise ratio should thus be advantageous using ^68^Ga-DOTANOC allowing for more straightforward and reproducible image interpretation, even in the hands of less experienced readers/physicians. A recent case study demonstrating avid ^68^Ga-DOTATOC uptake in the interventricular septum of a patient with convincing signs of CS underscores this notion [18].

Hence, the primary aim of the current study was to compare the diagnostic accuracy of ^18^F-FDG PET/CT and ^68^Ga-DOTANOC PET/CT for the diagnosis of CS. A secondary aim was to compare the reproducibility of image interpretations by comparing inter-observer variability for the two radiotracers. We hypothesized that ^68^Ga-DOTANOC PET/CT would outperform ^18^F-FDG PET/CT on both aims.

## Research design and methods

### Subjects

Nineteen patients with suspected cardiac sarcoidosis were prospectively enrolled from the Department of Respiratory Diseases or the Department of Cardiology at Aarhus University Hospital. Volunteers had either biopsy proven or clinically suspected sarcoidosis and all exhibited signs of cardiac involvement (arrhythmias or chronic heart failure). Retrospective reviews of patient files revealed that the clinical suspicion of sarcoidosis was later withdrawn in three patients (patients no. 9, 12, and 13—see Table 1).

**Table 1:** Patient characteristics and clinical presentation

Methotrexate	Age	Sex	Immunosuppressive therapy	ECG/Holter	ECHO	MRI	Extra-cardiac sarcoidosis site/biopsy	JMHW criteria fulfilled?	Major JMHW criteria	Minor JMHW criteria
1	41	M	Methotrexate 20 mg/weekly, prednisolone 7.5 mg/daily	SR	ND	No	Pulmonary/med. LN	No	None	None
2	54	M	None	SR, PVC, RBBB	Normal	Yes, anterior late enhancement	Pulmonary/clinical diagnosis	No	None	Non-sustained VT, MRI with anterior late enhancement
3	45	F	None	SR, PVC, RBBB	Anterior, septal and apical hypokinesia	No (pacemaker)	Kidney/kidney biopsy	Yes	Decreased LVEF	Left ventricular hypokinesia, VT, perfusion defects on scintigraphy
4	46	F	None	SR, PVC	Basal, lateral aneurism	No (pacemaker)	Pulmonary/bronchial biopsy	Yes	Decreased LVEF	Inferoseptal aneurism, perfusion defect on 82Rb PET, VT
5	37	M	None	3rd degree AV-block, ventricular escape rhythm	Normal LVEF, borderline right ventricular hypertrophy	Yes, septal late enhancement, thickening of the right ventricle	Pulmonary/clinical diagnosis	Yes	3rd degree AV-block	MRI with delayed enhancement in the septum + thickening of the right ventricle
6	59	M	None	SR, inferior Q-wave	Normal	Yes, no significant findings	Pulmonary/clinical diagnosis	No	None	PVC, RBBB
7	65	M	None	SR, RBBB, PVC	Normal	No (claustrophobia)	Pulmonary/med. LN	No	None	PVC, RBBB
8	36	F	None	SR, PVC	Normal	No	Pulmonary/splenic biopsy	No	None	PVC
9	59	M	None	SR, 1st degree AV-block, ST-depression	Dilated atrial cavities	Yes, basal and posterior late enhancement	None (sarcoidosis diagnosis withdrawn)	No (sarcoidosis diagnosis withdrawn)	Decreased LVEF	Left ventricular hypokinesia, MRI with late enhancement
10	60	F	None	SR	Discrete aortal insufficiency	No (pacemaker)	Skin/dermal biopsy	No	3rd degree AV-block	VT
11	64	M	Prednisolone 5 mg/daily	SR	Global hypokinesia	Yes, no significant findings	Pulmonary/cervical LN	No	Decreased LVEF	PVC
12	51	F	None	Pacemaker rhythm	Normal	No (pacemaker)	None (sarcoidosis diagnosis withdrawn)	No	3rd degree AV-block	
13	33	M	None	SR, ST-depressions in V5-V6	Global hypokinesia	Yes, dilated ventricles, no late enhancement	None (sarcoidosis diagnosis withdrawn)	No	Advanced 2nd degree AV-block, depressed LVEF	VT
14	58	M	Methotrexate 10 mg/weekly	SR	Normal	Yes, normal	Pulmonary/med. LN	No		
15	67	M	Prednisolone 5 mg/daily	Atrial flutter	Discrete hypertrophia, normal LVEF	No	Multiorgan/dermal biopsy	No		
16	46	F	Methotrexate 5 mg/weekly	SR	Normal	No	Pulmonary/med. LN	No		
17	39	M	None	SR	Normal	Yes, normal	Pulmonary/clinical diagnosis	No		
18	64	M	None	SR, LBBB	Discrete hypokinesia	No (nephropathy)	Pulmonary/skin	No	Decreased LVEF	Discrete hypokinesia of the septum
19	66	M	None	SR	Normal	No	Pulmonary/bronchial biopsy	No		

*Abbreviations*: *DE-MR* delayed enhancement magnetic resonance, *ECG* electrocardiogram, *ECHO* echocardiography, *EF* ejection fraction, *FEV1* forced expiratory volume, *HR-CT* high-resolution computed tomography, *JMHW* Japanese Ministry of Health and Welfare, *LV* left ventricle, *LBBB* left bundle branch block, *med. LN* mediastinal lymph node, *PVC* premature ventricular contractions, *RBBB* right bundle branch block, *SR* sinus rhytm, *VT* ventricular tachycardia

### Ethics, consent, and permissions

All volunteers received oral and written information concerning the study prior to giving written, informed consent. The protocol was approved by the Local Ethical Scientific Committee (1-10-72-200-12), registered at ClinicalTrials.gov (NCT01729169), and performed in accordance with the Helsinki Declaration II.

### PET acquisition

Patients were scanned with two PET radiotracers within 7 days: ^18^F-FDG and ^68^Ga-DOTANOC. All PET/CT studies were performed using the same Siemens Biograph 64 scanner (Siemens, Germany). Before each PET study day, patients were instructed to refrain from eating starch-containing foods and were instructed to fast from 8 pm the day before the PET scan. Average fasting time was 15 h. PET data acquisition started 60 (^18^F-FDG) and 90 (^68^Ga-DOTANOC) minutes after injection of 4 MBq/kg ^18^F-FDG and 3 MBq/kg ^68^Ga-DOTANOC, respectively. Acquisition time was 3 min/bed position. Non-gated CT imaging was used for attenuation correction. ^18^F-FDG PET scans were only performed if blood glucose was below 7 mmol/liter (126 mg/dl).

### Reference standard

In the absence of a validated histopathological reference standard, we opted for a combination of the revised CS criteria from 2006 laid out by the Japanese Ministry of Health and Welfare combined with clinical follow-up. During the clinical follow-up, electronic medical records were reviewed and any new arrhythmia, worsening of cardiac function, or morphological change assessed by echocardiography was considered indicative of cardiac sarcoidosis irrespective of the status at initial PET scan.

### Expert reviews and final PET diagnosis

Cardiac ^18^F-FDG PET images are difficult to review for signs of cardiac sarcoidosis. We therefore had four expert nuclear medicine physicians (P.B., A.H., S.K., and A.D.) review all studies enabling us to report both diagnostic accuracy of the two radiotracers and inter-observer variability. Readers were blinded to clinical data, biochemical tests, and results from other imaging modalities (US and MRI) and performed the image reviews in a random order. Majority decisions were used to decide patterns of ^18^F-FDG uptake and final PET diagnosis. For both radiotracers, PET scan results were classified as positive, negative, or inconclusive. For calculation of diagnostic accuracy, cases in which the majority decision was inconclusive were considered negative.

### ^18^F-FDG PET/CT image analysis

Reviewers were first asked to visually classify ^18^F-FDG images into four patterns: “none,” “diffuse,” “focal,” or “focal on diffuse,” where the none and diffuse patterns were considered physiological. Next, in case of focal and focal on diffuse patterns, reviewers were asked to consider whether these were thought to represent a normal physiological pattern (which is often seen in incomplete suppression of physiological cardiac ^18^F-FDG uptake) or if the uptake was indicative of CS. In this qualitative assessment, readers were asked to classify the images as “CS,” “no CS,” or “inconclusive”. Thus, the final ^18^F-FDG PET/CT diagnosis was purely qualitative.

For semiquantitative measurements, ROIs (circular with a radius of 1 cm) were placed in suspected CS lesions and SUVmax values and anatomic localization was recorded to compare anatomical precision of the image review.

### ^68^Ga-DOTANOC PET/CT image analysis

^68^Ga-DOTANOC is not accumulated in the cardiac region in healthy subjects and consequently, any uptake in the myocardium above blood pool activity was considered pathological. All identified areas of focally increased ^68^Ga-DOTANOC activity were recorded, and SUVmax values in individual lesions were measured.

## Statistics

Patient demographics and baseline characteristics are summarized as mean ± SD for continuous variables and frequencies for dichotomous or ordinal variables. Inter-observer agreement on the diagnosis of CS and patterns of ^18^F-FDG uptake was evaluated by estimating the overall agreement and the kappa statistic for multiple raters (experts) per subject (patient), a method put forward by Fleiss in 1971 [19]. Overall agreement on final diagnosis using Fleiss’ kappa is an estimate of the probability with which two randomly selected experts will agree on the diagnosis in a randomly selected patient. A kappa value of 0 indicates agreement equivalent to that expected by chance, negative values indicates agreement even lower than that. Values between 0 and 0.4 represent poor agreement, and values between 0.4 and 0.75 represent fair agreement and values above 0.75 represents excellent agreement.

Sensitivity, specificity, positive predictive value, negative predictive value, and diagnostic accuracy were calculated for both radiotracers based on the majority decision as outlined above.

## Results

### Characteristics of study subjects

Overall, 19 patients (male to female ratio 12:7, age (median, range) 54 years (33–67)) with suspected CS were prospectively enrolled from September 2012 to December 2014. Patient characteristics are detailed in Table 1. Median follow-up time was 17 months (range 4–31). One patient died from dilated cardiomyopathy 10 months after the PET scans. The remaining 18 were either completely discharged from any regular contacts with the outpatient clinic or in stable conditions at the time of the image reviews.

### Patients with cardiac sarcoidosis (reference standard)

In all, three patients fulfilled the JMHW criteria for CS (see Table 1). By comparison with the entire group of patients, they were younger (age 37–46) and women were over-represented (male to female ratio 1:2). All three patients were treated with high-dose corticosteroids for 6 months after which corticosteroid dose was reduced according to clinical presentation. All three patients had pacemakers implanted after the diagnosis of CS was established.

### Cardiac ^18^F-FDG PET—agreement on uptake patterns

**Fig. 1:**
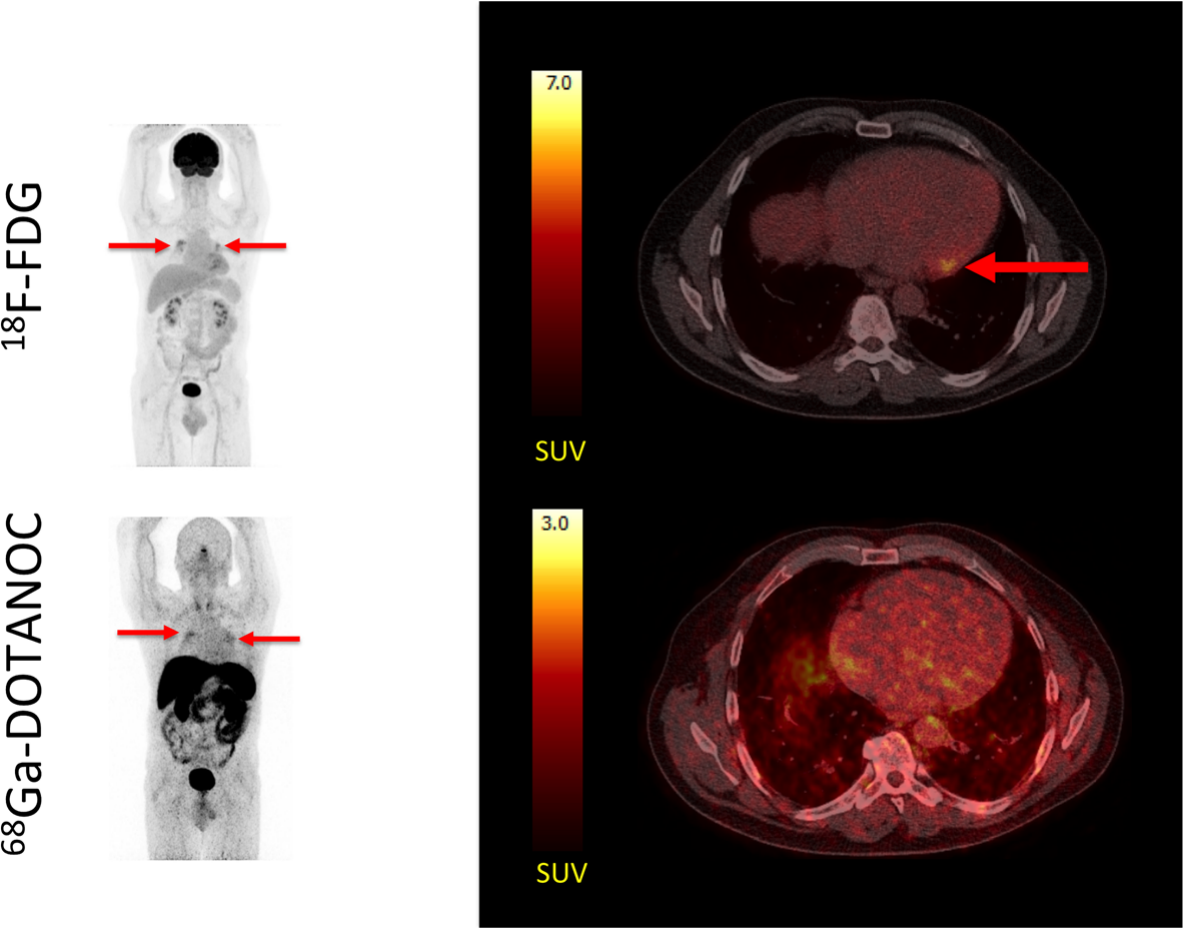
Typical ^18^F-FDG PET/CT false positive for cardiac sarcoidosis. *Left panel*: maximum intensity projections (MIPs) of patient no. 14. scanned after injection of ~370 MBq ^18^F-FDG and ~300 MBq ^68^Ga-DOTANOC. *Red arrows* denote ^18^F-FDG uptake in hilar lymph nodes (sarcoidosis). The hilar sarcoidosis is clearly visible on both ^18^F-FDG PET and ^68^Ga-DOTANOC PET. *Right panel*: transaxial slices of the cardiac region with ^18^F-FDG (*top*) uptake in the basal lateral wall of the myocardium (SUVmax 7.4). ^68^Ga-DOTANOC (*bottom*) images show uniform activity in the entire cardiac region (myocardium and blood pool) with no areas of focal uptake effectively ruling out CS. Notice the different SUV scales for the two radiotracers

**Fig. 2:**
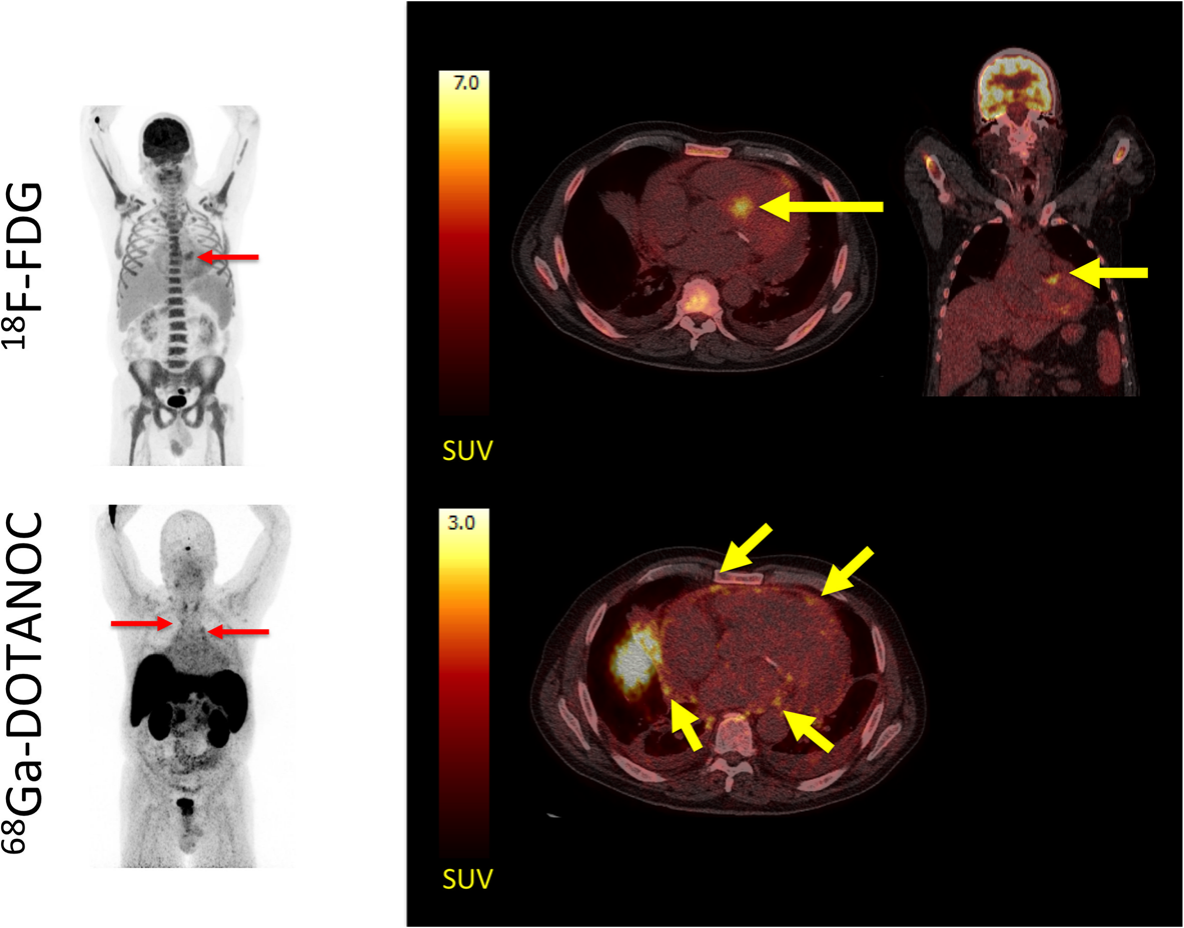
^18^F-FDG PET/CT false positive for cardiac sarcoidosis. *Left panel*: MIPs of patient no. 15 scanned after injection of ~370 MBq ^18^F-FDG and ~300 MBq ^68^Ga-DOTANOC. There was avid an ^18^F-FDG uptake in all areas of the bone marrow and the spleen indicating long-term infection. A few lymph nodes are visible in the upper mediastinum on the ^68^Ga-DOTANOC scan. *Right panel*: transaxial and coronal slices of the cardiac region revealed avid ^18^F-FDG uptake in the area around the aortic ostium (*yellow arrows*) which is often seen in aortic valve sclerosis, but can also be mistaken for uptake in a sarcoid lesion (SUVmax 7.6). Clear calcifications are seen around the posterior valve on the low-dose CT. On the ^68^Ga-DOTANOC scan (*bottom*), there is no activity above background in the myocardium. By contrast, activity is increased in the pericardium (*yellow arrows*) and some pericardial fluid is visible indicating pericarditis

**Fig. 3:**
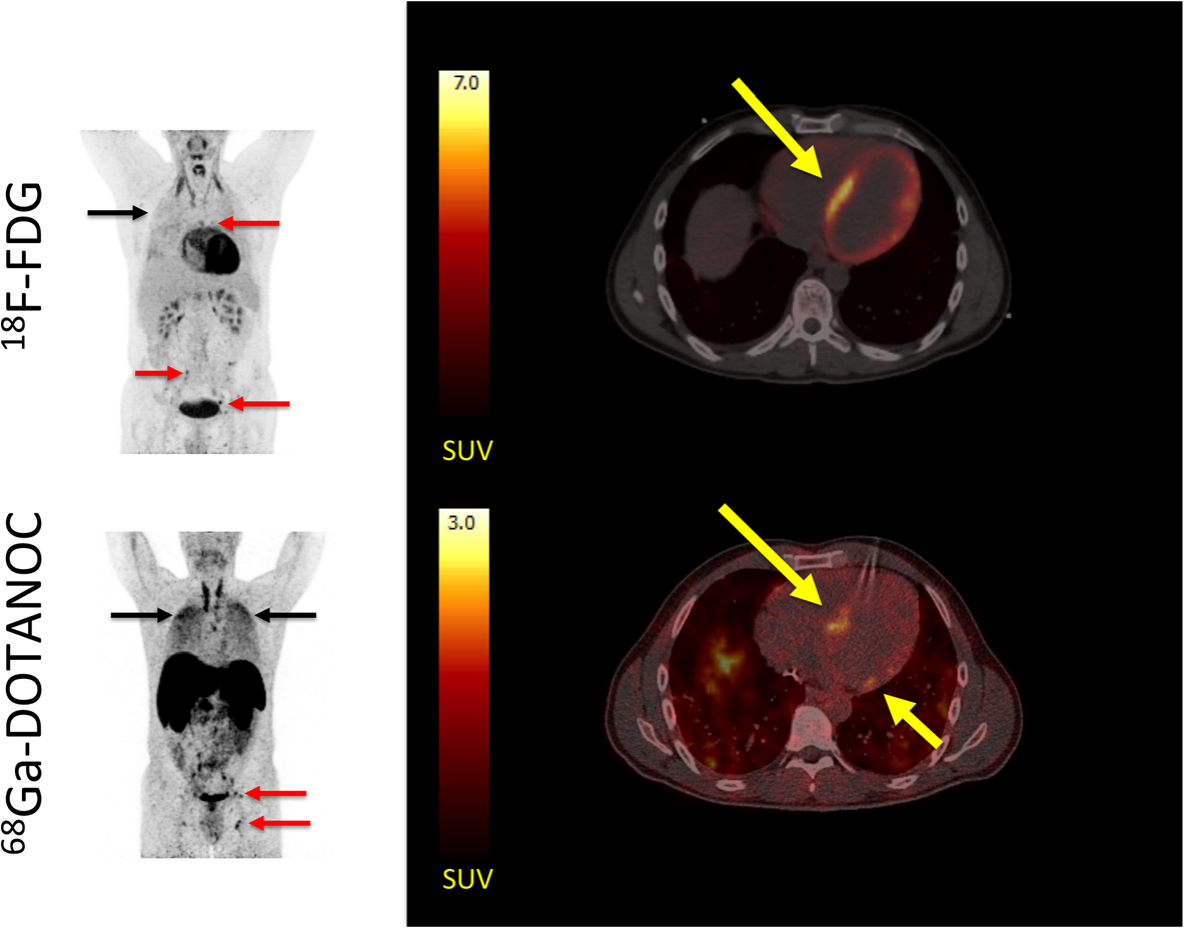
Patient with CS in which the ^18^F-FDG PET/CT was inconclusive due to insufficiently suppressed physiological ^18^F-FDG uptake by the myocardium. *Left panel*: MIPs showing patient no. 5 with dilated cardiomyopathy and multiple ^18^F-FDG and ^68^Ga-DOTANOC avid lymph nodes (*red arrows*) both above and below the diaphragm. In addition, there is massive and diffusely increased activity in the lung parenchyma (*black arrows*) representing active pulmonary sarcoidosis. *Right panel*: transaxial slices of the cardiac region reveal a focal on diffuse pattern of ^18^F-FDG uptake (*top*) raising suspicion of cardiac involvement (SUVmax 21 in the septum). However, the image was rated inconclusive by a majority of expert reviewers. By contrast, all reviewers rated the ^68^Ga-DOTANOC uptake (SUVmax 2.8, target-to-background 3.04) in the septum pathological (*bottom*). The patient was treated with corticosteroids and recovered

The four expert reviewers were asked to categorize cardiac ^18^F-FDG uptake according to the patterns suggested by Ishimaru et al. [20]. Somewhat to our surprise, complete suppression of cardiac ^18^F-FDG uptake (none pattern) was accomplished in only 2/19 patients (see Additional file 1: Table S1). In 11/19 patients, the majority decision on ^18^F-FDG pattern was diffuse indicating at least some presumably insulin-independent ^18^F-FDG uptake. In 2/3 patients with CS as determined by the reference standard, ^18^F-FDG uptake was focal on diffuse, whereas it was classified as diffuse in the last patient, who had severely dilated cardiac cavities (see Fig. 3). ^18^F-FDG uptake was classified as focal on diffuse or focal in four patients who did not meet the JMHW criteria (patients no. 7, 9, 14, and 15) (see Figs. 1 and 2 for example). One of these patients died of non-cardiac-related disease 10 months after the PET scans (and never had the sarcoidosis diagnosis confirmed), and the three remaining were all discharged without high-dose corticosteroid therapy (since CS was ruled out by JMHW criteria). At follow-up, these three patients were all doing well and had no signs of active sarcoidosis. Overall agreement between the reviewers on patterns of ^18^F-FDG uptake was 0.72 (Fleiss’ kappa) indicating fair agreement on the patterns of FDG-uptake.

### Cardiac ^18^F-FDG PET—agreement on diagnosis

Based on the visual inspection of cardiac ^18^F-FDG uptake, the expert reviewers were asked to assess whether the ^18^F-FDG PET scan indicated CS, no CS, or was inconclusive. In 11/19 patients, the majority found the ^18^F-FDG scan inconclusive; in 5/19 patients, the diagnosis was “no sign of sarcoidosis”; and in 3/19 patients, the majority decision was “cardiac sarcoidosis” (see Additional file 2: Table S2). Of the three patients with CS using the JMHW criteria as the standard of truth, only one patient was classified as such by majority decision on the ^18^F-FDG PET. The remaining two patients with CS had inconclusive scans. False positive scans were seen in patient 14, where ^18^F-FDG uptake was seen in the basal lateral wall (see Fig. 1), and in patient no. 15 (see Fig. 2), where ^18^F-FDG uptake near the aortic ostium was observed. This is often seen in sclerotic valvulopathy. Overall agreement on the diagnosis of CS by ^18^F-FDG PET was 0.27 (Fleiss’ kappa) indicating poor agreement.

### Cardiac ^18^F-FDG PET—diagnostic accuracy

For calculation of diagnostic accuracy, inconclusive scans were considered negative. Sensitivity of ^18^F-FDG cardiac PET for diagnosing CS was 33 % (1/3), specificity was 88 % (14/16), PPV was 33 % (1/3), NPV was 88 % (14/16), and diagnostic accuracy was 79 %. The binary classification data for ^18^F-FDG is summarized in Table 2.

**Table 2:** Binary classification characteristics of ^18^F-FDG

		CS diagnosis (JMHW criteria)		
		Positive	Negative	
FDG diagnosis	Positive	1	2	3
	Negative/inconclusive	2	14	16
		3	16	19

### Cardiac ^68^Ga-DOTANOC PET—agreement on diagnosis

**Fig. 4:**
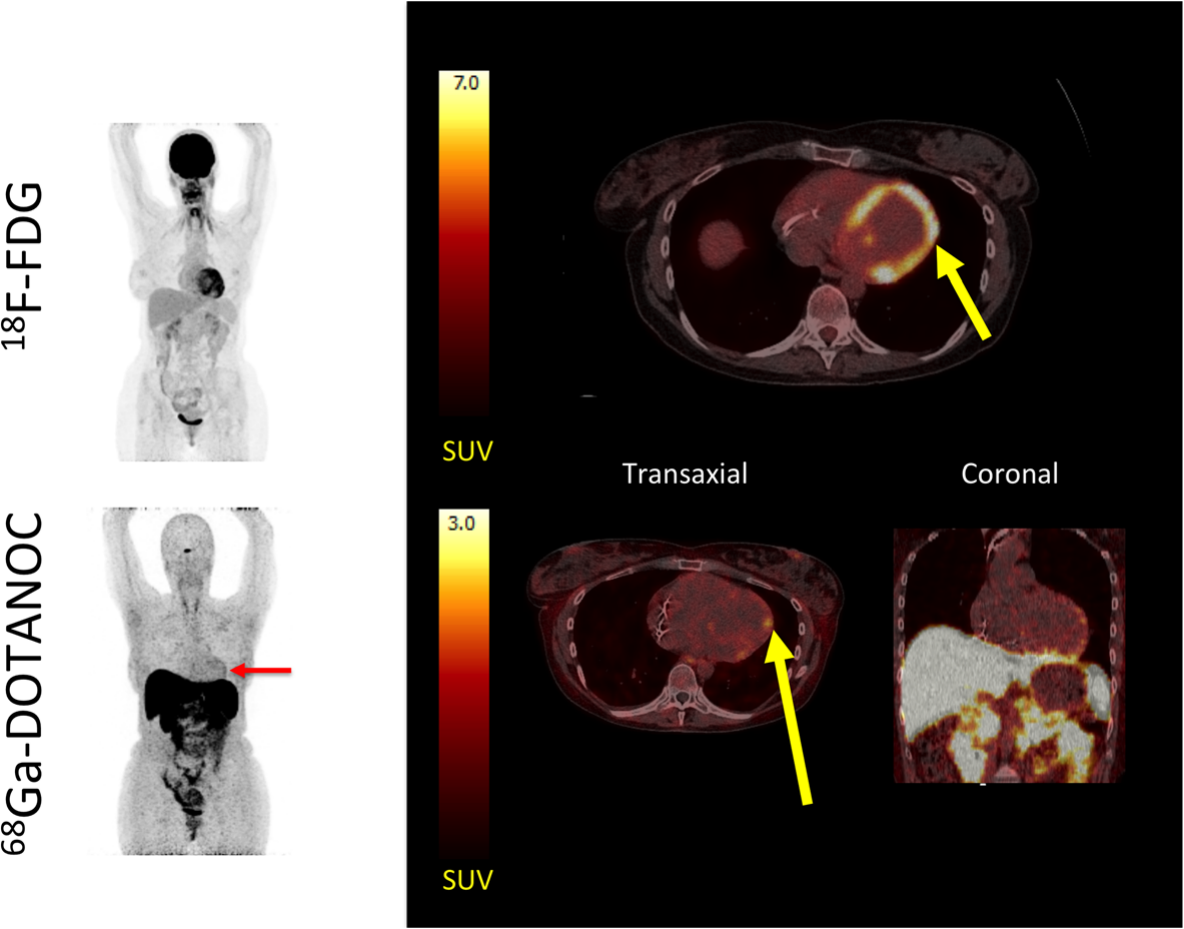
Patient with CS. ^18^F-FDG PET/CT images were rated as inconclusive due to insufficiently suppressed physiological ^18^F-FDG uptake. *Left panel*: MIPs showing ^18^F-FDG PET/CT (*upper*) and ^68^Ga-DOTANOC PET/CT (*lower*) of patient no. 3. ^68^Ga-DOTANOC accumulation can be seen in the anterior and lateral wall (*red arrow*). *Right upper panel*: transaxial slices of ^18^F-FDG showing focal on diffuse ^18^F-FDG uptake considered inconclusive by a majority of readers. *Right lower panel*: ^68^Ga-DOTANOC accumulation (*yellow arrow*) is seen in the anterolateral and lateral wall (SUVmax 2.35, target-to-background 1.56). In the coronal view, it is evident that spill-in activity from the liver may obscure inferior lesions

**Fig. 5:**
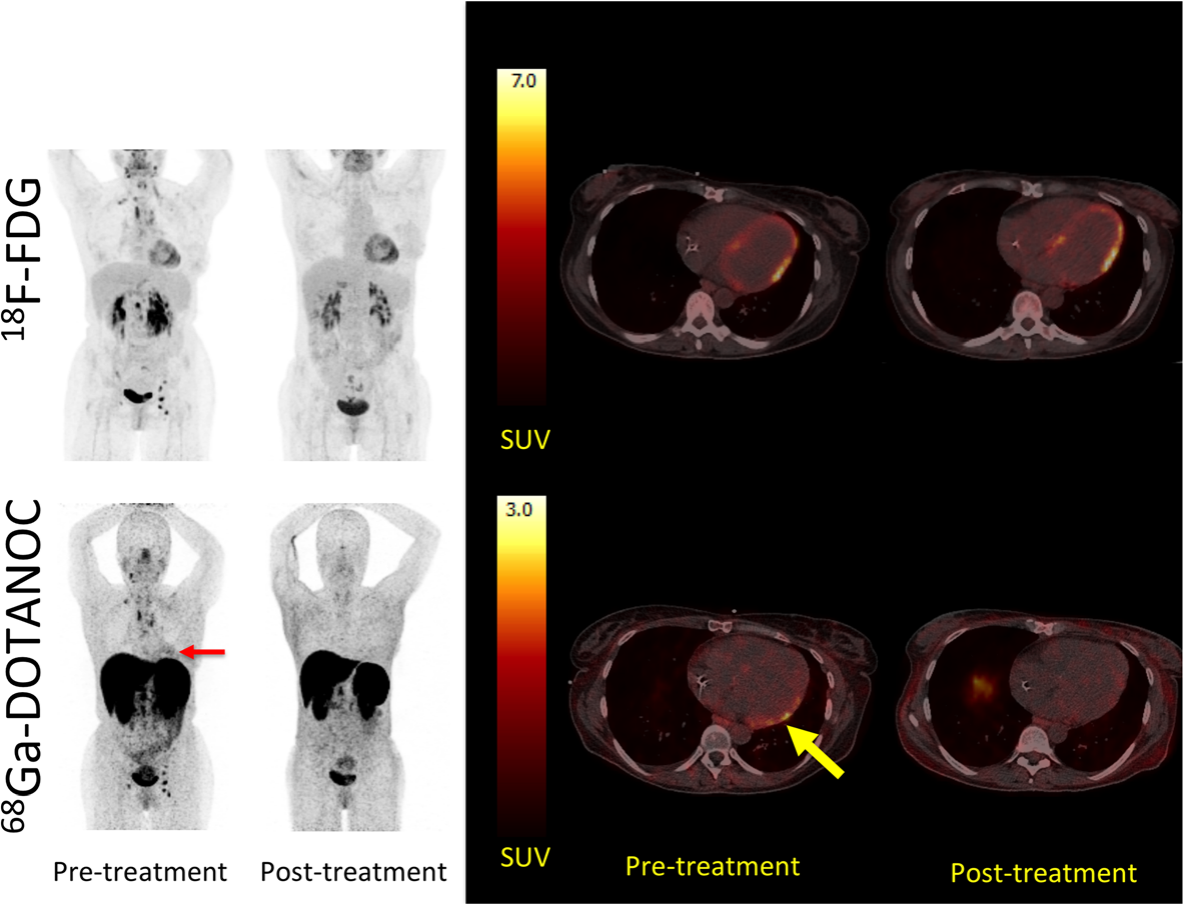
The effects of corticosteroid treatment on ^18^F-FDG and ^68^Ga-DOTANOC uptake in the cardiac region. *Left panel*: patient no. 4 (with CS according to the reference standard) was dual-tracer scanned before treatment with corticosteroids and after 6 months of high-dose prednisolone treatment (initially 50 mg tapering off to 37.5 mg). At diagnosis, there were multiple ^18^F-FDG and ^68^Ga-DOTANOC avid lymph nodes on both sides of the diaphragm as well as accumulation of ^68^Ga-DOTANOC in the cardiac region (*red arrow*) (SUVmax 2.64, target-to-background 2.54). The patient was rated as positive for CS on the ^68^Ga-DOTANOC PET/CT. *Right panel*: transaxial slices of the same patient where ^18^F-FDG uptake in the myocardium (*upper row*) is similar during both scans (focal on diffuse) whereas the ^68^Ga-DOTANOC accumulation (*lower row*) in the basal inferolateral wall (*yellow arrow*) is completely abolished after treatment

As anticipated, agreement among reviewers was better for the ^68^Ga-DOTANOC scans owing primarily to significantly fewer scans categorized as inconclusive. Thus, no scans were inconclusive by majority decision and more scans were categorized similarly by all reviewers (see Additional file 3: Table S3). 3/19 patients were classified as CS by majority decision (see Figs. 3, 4, and 5). However, overall agreement assessed by Fleiss’ kappa was only fair (0.46) which is probably attributable to the rather modest SUVmax values recorded in the lesions (ranging between 2.5 and 2.8).

### Cardiac ^68^Ga-DOTANOC PET—diagnostic accuracy

Using the JMHW criteria for CS, ^68^Ga-DOTANOC correctly classified all patients and thus had a diagnostic accuracy of 100 %. The binary classification data for ^68^Ga-DOTANOC is summarized in Table 3.

**Table 3:** Binary classification characteristics of ^68^Ga-DOTANOC

		CS diagnosis (JMHW criteria)		
		Positive	Negative	
DOTANOC diagnosis	Positive	3	0	3
	Negative	0	16	16
		3	16	19

### Effects of corticosteroid therapy on cardiac ^18^F-FDG and ^68^Ga-DOTANOC uptake

Follow-up scans using both tracers were available for patient no. 4 (see Fig. 5) diagnosed with CS. Interestingly, the focal on diffuse ^18^F-FDG uptake pattern was completely unaltered by corticosteroid therapy whereas all ^18^F-FDG uptake in affected lymph nodes above and below the diaphragm was normalized. The focal on diffuse cardiac ^18^F-FDG uptake pattern could thus safely be considered physiological.

By contrast, pre-therapeutic ^68^Ga-DOTANOC uptake was only increased in a small area in the basal lateral area, where US showed an aneurism. This pathological ^68^Ga-DOTANOC uptake was completely abolished by corticosteroid therapy, and no uptake above blood pool level was evident in the myocardium. As for ^18^F-FDG, ^68^Ga-DOTANOC uptake by affected lymph nodes was completely absent after therapy indicating an excellent overall response.

## Discussion

In this proof-of-concept study, we have shown that inflammation in the form of sarcoid granulomas takes up ^68^Ga-DOTANOC. By consensus decision, the diagnostic accuracy of ^68^Ga-DOTANOC in diagnosing CS was 100 %—with the caveat, which inter-observer agreement was only fair owing to the modest tracer accumulation in sarcoid lesions. Most importantly, ^68^Ga-DOTANOC PET/CT images were only rarely classified as inconclusive reflecting the advantageous signal-to-noise ratio in the cardiac region. By contrast, even though expert readers could agree on the cardiac ^18^F-FDG pattern, insufficient suppression (15 h fasting) of physiological cardiac ^18^F-FDG uptake resulted in a large proportion of inconclusive ^18^F-FDG PET/CT scans. This study therefore also supports recent studies [21] that underscore the importance of more rigorous pre-scan regimens involving either high-fat/low-carbohydrate or Intralipid/heparin infusions.

### Cardiac ^18^F-FDG PET/CT

Despite a growing body of evidence that supports the use of cardiac ^18^F-FDG PET/CT to diagnose CS, numerous problems regarding physiological myocardial ^18^F-FDG uptake still persist. This becomes particularly evident when cardiac ^18^F-FDG PET/CT is implemented in an everyday clinical practice. First, patient preparations are not straightforward: In order to minimize physiological ^18^F-FDG uptake by the cardiomyocytes, lipid levels must be raised while insulin levels should be suppressed. This is typically achieved by a combination of fasting, diet interventions, and infusions of Intralipid and heparin. However, fasting for the recommended 18 h [22] requires skipping at least one major meal which likely reduces overall compliance. In addition, as few as ~10 % of patients may obtain complete suppression of physiological cardiac ^18^F-FDG uptake [23, 24] even if the fast is adhered to. Patient preparations become even more complicated, when the fast must be combined with a starch-poor diet to produce optimal results [12, 13] or when heparin and lipid emulsions (Intralipid) are injected just prior to or during the scan in order to release more fatty acids into the circulation [25]. These attempts to force the cardiac region to rapidly switch from glucose to lipid fuels for oxidation have produced mixed results with our own combined Intralipid/heparin/somatostatin infusion failing to significantly suppress cardiac ^18^F-FDG uptake in patients with suspected CS [26]. To sum up, laborious pre-scan diet and infusion protocols still result in a high probability of insufficiently suppressed physiological ^18^F-FDG uptake. Second, when physiological cardiac ^18^F-FDG uptake is not suppressed, it is frequently heterogenous [14, 27] with basal lateral uptake exceeding uptake in other regions [28]. Calcifications of the aortic valves [29], atrial fibrillation [30], and coronary artery disease [31] may also produce focally or heterogeneously increased ^18^F-FDG uptake not related to sarcoidosis. This was most likely the case in patient no. 15, where focal accumulation of ^18^F-FDG adjacent to sclerotic aortic valves was taken as evidence of CS, and in patient no. 14 where a single focus of ^18^F-FDG was present in the basal lateral wall. These pitfalls to visual interpretation of cardiac ^18^F-FDG PET/CT have prompted a series of semiquantitative approaches ranging from SUVmax cut-offs [25, 32] to analyses of cardiac ^18^F-FDG heterogeneity [12]. Analytic improvements are likely to improve diagnostic accuracy but require such strict standardization of patient preparation, scan protocols, and image reconstruction that separate studies in each PET centre are warranted to establish relevant threshold values.

Somewhat disappointingly, diagnostic accuracy of ^18^F-FDG PET/CT was rather low in this study when compared with previously published data. However, geography may to some extent explain this. Thus, the reported frequency of cardiac involvement in patients with sarcoidosis ranges from 58 % in patients of Japanese origins [33] to 16 % in Scandinavian Caucasians with ECG changes [34]. Focal or heterogenous ^18^F-FDG uptake in patients with CS caused entirely by physiological glucose uptake and not by accumulation in inflammatory cells should thus be categorized as false positive on a lesion basis, but could in Japanese patients with sarcoidosis be true positive on a patient basis (since more than half of all Japanese patients probably have CS at some stage). This misclassification is much less likely to occur in Europe or North America, where the prevalence of CS in sarcoidosis patients is markedly lower. The excellent specificity of ^18^F-FDG PET/CT reported by Okumura [35] and Ishimaru [20] who conducted studies in Japan appear to support this notion. Also in line with this, we observed focal on diffuse ^18^F-FDG uptake in CS patient no. 4 on the initial scan and could therefore classify the scan as positive for CS. However, the pathological ^68^Ga-DOTANOC accumulation was located elsewhere and completely disappeared with corticosteroid therapy, whereas the post-therapeutic ^18^F-FDG uptake pattern was unaltered (see Fig. 5). In our opinion, this indicates normal physiology despite heterogenous ^18^F-FDG uptake. The patient was therefore false positive on a lesion basis but true positive on a patient basis.

### ^68^Ga-DOTANOC PET/CT

The use of SSTR-targeted radiotracers to assess sarcoidosis disease activity is not a novel idea. Giant cells, epithelioid cells, and lymphocytes constitute the bulk of active inflammatory cells in sarcoid granulomas and express SSTRs abundantly. Thus, Kwekkeboom et al. [36] were able to visualize 97 % of known sarcoid lesions in 46 patients using 111In-labeled pentetreotide (octreotide), a SSTR analogue visualized by gamma camera technique. These results were largely reproduced in a later but smaller study, where 83 % of clinically involved sarcoidosis sites were identified [37]. No uptake was observed in the cardiac region in either study. This may at least partly be explained by the poor spatial resolution of octreotide scintigraphies but also by the composition of patient cohorts that did not include patients with suspected CS.

The introduction of ^68^Ga-labeled SSTR ligands may change this. ^68^Ga is a generator-produced PET isotope that has been used to label a range of SSTR-targeting ligands developed to diagnose and monitor [38] neuroendocrine tumors. ^68^Ga-DOTANOC PET/CT binds to SSTR2, 3, and 5 and has better spatial resolution than octreotide scintigraphies. In addition, PET and CT images may be fused allowing for better anatomical localization of any suspected lesion. Somewhat surprisingly, we are unaware of any previous studies using ^68^Ga-labeled SSTR ligands to diagnose CS. Only one case study of a patient with suspected CS has recently been published in which ^68^Ga-DOTATOC was avidly taken up by the interventricular septum indicating cardiac involvement [18]. In addition to this case study, the same group has published evidence of ^68^Ga-DOTATOC accumulation in post-infarction myocarditis as well as in pericarditis further strengthening the point that SSTR ligands bind to inflammatory cells [39].

Using ^68^Ga-DOTANOC PET/CT, we were able to correctly identify all three patients who met the revised JMHW criteria for CS. Furthermore; we were able to rule out cardiac involvement in the remaining patients. As expected, we frequently observed avid ^68^Ga-DOTANOC accumulation in multiple involved lymph nodes (see figures), which served as a positive control of active sarcoidosis for the visual assessment of cardiac involvement. The rather low prevalence of CS observed in this patient cohort is comparable to that observed by Ishimaru et al. (5/32) [20] but somewhat lower than that reported in a large study by Blankstein et al. (34/118) [40]. In our opinion, this reflects different patient recruitment strategies since 18 patients from the Blankstein study had known cardiac sarcoidosis at the time of their PET scan.

Importantly, we observed no new arrhythmias or worsening of left ventricular function in patients classified as negative for ^68^Ga-DOTANOC accumulation in the cardiac region (excluding the patient who died of other causes) and NPV was therefore excellent. However, ^68^Ga-DOTANOC SUVmax values observed in suspected lesions were only modest compared to ^18^F-FDG SUVmax values (which were as high as 21 in patient no. 5). Thus, it is of great importance that there is no physiological ^68^Ga-DOTANOC accumulation in the myocardium.

Finally, although a diagnostic accuracy of 100 % was seen for ^68^Ga-DOTANOC, this was only obtained by majority decision, and some inter-rater disagreement was seen for ^68^Ga-DOTANOC. Although all raters were experts with years of PET/CT experience, it is likely that some of this disagreement was caused by the raters being unaccustomed to evaluating the cardiac region on ^68^Ga-DOTANOC images, since this is not part of clinical routine. In contrast, all raters were experienced in evaluating cardiac FDG uptake, which was reflected by the relatively high agreement on FDG uptake patterns (Fleiss kappa 0.72). We believe that inter-rater agreement on pathological cardiac DOTANOC uptake probably can be substantially improved by exposing the raters to a ^68^Ga-DOTANOC training dataset of verified CS cases. This strategy was not available to us, since only three patients had active CS and expert readers therefore could not undergo a regular training session with demonstration of CS image characteristics (^68^Ga-DOTANOC) for fear that individual patients would subsequently be recognizable.

### Limitations

Several limitations apply to the current study. First, the prevalence of CS was low with only three patients fulfilling the JMHW criteria for CS. Should the study have been adequately powered, it would have required a very large study population (approximately 400) [41], which is impossible for practical and ethical reasons in radiotracer studies. It is therefore not possible to draw firm conclusions as to the superiority of ^68^Ga-DOTANOC over ^18^F-FDG PET/CT in detecting CS. The findings from this pilot study should therefore serve more as proof-of-concept than a definite study of diagnostic accuracy.

Second, only active inflammatory cells express SSTRs and late-stage CS, which is characterized by fibrosis and scarring, is therefore unlikely to take up ^68^Ga-DOTANOC. In addition, ^68^Ga-DOTANOC is taken up by inflammatory cells irrespective of their causative factor and is therefore not suited to distinguish between sarcoidosis and, e.g., infective myositis or endocarditis. However, this caveat applies equally to ^18^F-FDG PET.

Third, the absence of concurrent cardiac MRI scans of all patients in the present study is also a major limitation. Areas of active inflammation as well as fibrotic scars display slow wash-out of gadolinium and this late gadolinium enhancement (LGE) has been demonstrated to accurately predict the presence of cardiac sarcoidosis. Thus, Smedema et al. detected LGE in all 12 patients with cardiac sarcoidosis using the JMHW criteria as a reference standard. However, LGE may also be a non-specific finding in, e.g., dilated cardiomyopathy, and consequently, the specificity (78 %) and positive predictive value (55 %) of MRI was somewhat lower in that study [8]. Others have developed more elaborated MRI protocols involving T2-mapping which may increase the specificity of MRI for detecting CS [42]. In summary, cardiac MRI is an excellent first-line imaging modality in patients with suspected CS provided there are no contraindications to its use. Unfortunately, this may often be the case in patients with CS since advanced AV-blocks necessitating rapid implantation of a pacemaker is often a presenting symptom.

Fourth, the diagnostic accuracy of our ^18^F-FDG PET/CT scans was disappointingly low, which may relate to the pre-scan protocol we opted for. We did not attempt to control participating subjects’ diet with high-fat meals preceding the scan, nor did we co-infuse Intralipid and heparin immediately prior to the scan as advocated by others (reviewed in [21]).

## Conclusions

Cardiac sarcoidosis poses a diagnostic challenge with no single test available to correctly identify patients. We herein present data indicating that ^68^Ga-DOTANOC PET/CT can be used as an adjunct imaging modality in patients with suspected CS—preferably as an imaging substitute for the obsolete ^67^Ga citrate scintigraphy. In a small cohort of patients with suspected CS, cardiac ^68^Ga-DOTANOC PET/CT had excellent diagnostic accuracy and had the advantage over ^18^F-FDG PET/CT that no pre-scan fasts, diets, or infusions were required. However, since this was a pilot study involving only few cases of CS, the findings must be reproduced in a larger patient cohort. It should also be noted that solitary inferior lesions may be obscured by physiological activity in the liver and ventricle.

## Additional files

Additional file 1: Table S1.
^18^F-FDG uptake patterns. (DOCX 65.8 kb) Additional file 2: Table S2. Individual ^18^F-FDG ratings. (DOCX 65.8 kb) Additional file 3: Table S3. Individual ^68^Ga-DOTANOC ratings. (DOCX 64 kb)
