# Supplementary material for: A dual tracer 68Ga-DOTANOC PET/CT and 18F-FDG PET/CT pilot study for detection of cardiac sarcoidosis
Source: EJNMMI Res. 2016 Jun 17;6:52. doi: 10.1186/s13550-016-0207-6 (PMC4912521; doi:10.1186/s13550-016-0207-6)
Supplement: Additional file 3: Table S3. — Individual 68Ga-DOTANOC ratings. (DOCX 64 kb) [file 13550_2016_207_MOESM3_ESM.docx]

Supplementary table 3. Individual 68Ga-DOTANOC ratings

|  | Rater | | | |  |  |
| --- | --- | --- | --- | --- | --- | --- |
| Subject | 1 | 2 | 3 | 4 | Majority decision | Diagnosis (JMHW) |
| 1 | 0 | 0 | 0 | 0 | No CS | No CS |
| 2 | 0 | 0 | 0 | 1 | No CS | No CS |
| 3 | 1 | 0 | 1 | 1 | CS | CS |
| 4 | 1 | 1 | 0 | 1 | CS | CS |
| 5 | 1 | 1 | 1 | 0 | CS | CS |
| 6 | 0 | 0 | 0 | 0 | No CS | No CS |
| 7 | 0 | 0 | 0 | 0 | No CS | No CS |
| 8 | 0 | 0 | 0 | 0 | No CS | No CS |
| 9 | 0 | 0 | 0 | 0 | No CS | No CS |
| 10 | 0 | 0 | 0 | 0 | No CS | No CS |
| 11 | 0 | 0 | 0 | 0 | No CS | No CS |
| 12 | 0 | 0 | 0 | 0 | No CS | No CS |
| 13 | 0 | 0 | 0 | 0 | No CS | No CS |
| 14 | 0 | 0 | 0 | 0 | No CS | No CS |
| 15 | 0 | 0 | 0 | 0 | No CS | No CS |
| 16 | 0 | 0 | 0 | 0 | No CS | No CS |
| 17 | 0 | 0 | 0 | 0 | No CS | No CS |
| 18 | 0 | 0 | 0 | 0 | No CS | No CS |
| 19 | 0 | 0 | 0 | 1 | No CS | No CS |
| Fleiss’ kappa | | | | |  | 0.46 |

0 = No CS, 1 = CS, 2 = Inconclusive
